# Evaluating COVID-19 vaccine acceptance among parents in Saudi Arabia: a systematic review examining attitudes, hesitancy, and intentions

**DOI:** 10.3389/fpubh.2024.1327944

**Published:** 2024-03-22

**Authors:** Anwar A. Sayed

**Affiliations:** Department of Basic Medical Sciences, College of Medicine, Taibah University, Madinah, Saudi Arabia

**Keywords:** attitudes, COVID-19 vaccine, hesitancy, parents, public health, Saudi Arabia, systematic review, vaccines willingness

## Abstract

**Introduction:**

The COVID-19 pandemic, affecting adults and children equally, has caused significant disruption to countries worldwide, including Saudi Arabia. In Saudi Arabia, the fast preventative measures and mass vaccine enrollment were vital to contain the devastating impact of the pandemic. However, vaccine hesitancy, especially among parents toward vaccinating their children, was a significant obstacle to vaccine uptake.

**Methods:**

This systematic review followed PRISMA guidelines to assess parental willingness to vaccinate their children against COVID-19, determine the key determinants influencing such intention and attitudes, and underline the significant concerns and misconceptions regarding the vaccine among parents. The Joanne Briggs Institute (JBI) checklist for prevalence studies was used to assess included studies for risk of bias.

**Results:**

Twenty-three studies were included in this systematic review, representing a total of 20,926 participants, with over 66% of them were female. Over 37% of the participants were willing to vaccinate their children against COVID-19. Parents’ age, gender, level of education, and income were the main determinants of their intention to vaccinate their children. The parents’ main concerns were the potential vaccine side effects, safety, and efficacy. Major misconceptions about the COVID-19 vaccine included it being dangerous to children and that children are at lower risk of severe infection; hence, vaccines were not needed.

**Discussion:**

This seminal review provides insights to public health policymakers, which should be considered and taken together in light of other studies addressing parental vaccine hesitancy.

## 1 Introduction

The global battle against the COVID-19 pandemic has relied significantly on the rapid development and dissemination of vaccines to mitigate the impact of the virus. Like other countries, Saudi Arabia deployed a series of progressive measures to tackle the spread of the virus (1). It also initiated mass vaccination campaigns, which have proven effective in curbing the spread of COVID-19, reducing severe illness, and saving lives (2). Nevertheless, the success of these vaccination efforts is intrinsically tied to the willingness of individuals and, crucially, parents to accept and partake in the vaccination process. The existence and growth of vaccine hesitancy among parents present a multifaceted challenge that can hinder the achievement of herd immunity and prolong the pandemic.

So far, there is not an exact definition for vaccine hesitancy. However, it is often explained as a delay or refusal to accept vaccination despite its availability. Vaccine hesitancy is a complex issue influenced by many factors, including personal beliefs, societal contexts, cultural norms, and political climates (3, 4). Parental vaccine hesitancy, toward vaccinating their children, is of particular concern, as it holds the power to impact not only the health and well-being of children but also to lower overall vaccination rates, posing a significant barrier to ending the COVID-19 pandemic (5).

While vaccine hesitancy has been the subject of global scrutiny, it is essential to acknowledge that its underlying drivers and manifestations vary significantly across diverse regions and populations. Saudi Arabia, a nation shaped by its unique blend of cultural, religious, and healthcare influences, calls for a meticulous examination of parental vaccine hesitancy concerning COVID-19 vaccines.

This systematic review aims to offer an all-encompassing portrayal of the present state of parental vaccine hesitancy within the context of Saudi Arabia, with a specific emphasis on COVID-19 vaccines. The objectives of this systematic review are to evaluate the willingness of parents in Saudi Arabia to vaccinate their children against COVID-19, and to determine the key factors driving their vaccination intention. This is done by meticulously synthesizing and scrutinizing existing literature to underline the factors contributing to vaccine hesitancy among Saudi parents, elucidate the public health implications, and propose a framework for addressing this pressing concern. This research is both timely and imperative, as it can provide crucial insights into the design of targeted interventions and policies intended to enhance vaccine acceptance and coverage, thereby contributing significantly to the global campaign to control the COVID-19 infection.

## 2 Materials and methods

### 2.1 Search strategy and selection criteria

A systematic review was conducted following the Preferred Reporting Items for Systematic Reviews and Meta-Analyses (PRISMA) guidelines (5). The primary objective was to identify and analyze studies investigating parental vaccine hesitancy toward COVID-19 vaccines in Saudi Arabia. The search was conducted in major electronic databases, including PubMed, Scopus, and MEDLINE, from January 2020 to August 2023. The search strategy combined keywords related to COVID-19 vaccines (“COVID-19,” “vaccine”) with terms specific to parental vaccine hesitancy (“parents”) and Saudi Arabia (“Saudi Arabia”). The low number of search terms was intended to maximize the number of retrieved studies. As the systematic review addresses parental vaccine hesitancy against COVID-19, published studies about the topic included in the review were published between 2020 and 2023. However, no time-period restrictions were set during the literature search.

### 2.2 Inclusion and exclusion criteria

Studies were included if they met the following criteria: original research articles, included residents of Saudi Arabia, focused solely on parental vaccine hesitancy toward COVID-19 vaccines, written in English or Arabic, published between January 2020 and August 2023. Excluded studies that did not meet these criteria, such as review articles, editorials, or multinational studies, as they may not accurately reflect the drivers of vaccine hesitancy among Saudi populations.

### 2.3 Data extraction

Once the studies have been identified and refined, based on the criteria mentioned in the previous section, studies were easily retrieved as they were open-access articles. The studies were critically analyzed to extract the information required for primary and secondary outcomes. Information about the study locality, e.g., region/city, as the well as the participants’ genders, were recorded.

### 2.4 Risk of bias appraisal

For quality assurance purposes, the retrieved articles were assessed for bias using the Joane Briggs Institute (JBI) critical appraisal checklist for prevalence studies (6). Briefly, the JBI checklist consists of 9 questions addressing issues related to sampling studies’ population, sample size, the use of valid methodological and statistical tools, and response rate. Each item of the checklist can be labelled with Yes (to indicate the achievement of such measure), No (did not achieve the measure), or Unclear (which indicates lack of clarity on the achievement of the measure). The detailed breakdown of the appraisal of the included articles is described in Table 1.

**Table 1:** The JBI appraisal of the studies included in this systematic review.

No.	References	Q1	Q2	Q3	Q4	Q5	Q6	Q7	Q8	Q9	Overall appraisal
1	Temsah et al. (7)	Y	Y	Y	Y	Y	Y	Y	Y	UC	Include
2	Altulaihi et al. (8)	N	Y	Y	Y	Y	Y	Y	UC	Y	Include
3	Aedh (9)	Y	Y	Y	Y	Y	Y	Y	Y	UC	Include
4	Alzahrani and Alghamdi (10)	Y	Y	Y	Y	Y	Y	Y	Y	N	Include
5	Ennaceur and Almohaithef (11)	UC	UC	Y	Y	Y	Y	N	Y	Y	Include
6	Shaati et al. (12)	Y	Y	Y	Y	Y	Y	Y	Y	UC	Include
7	Al Saad et al. (13)	Y	Y	Y	Y	N	Y	Y	Y	UC	Include
8	Al-Khlaiwi et al. (14)	Y	UC	Y	Y	Y	Y	N	Y	UC	Include
9	Alghamdi (15)	Y	Y	Y	N	Y	Y	Y	N	UC	Include
10	Khan et al. (16)	Y	Y	Y	Y	Y	Y	UC	Y	Y	Include
11	Alhuzaimi et al. (17)	Y	UC	N	Y	Y	Y	Y	Y	UC	Include
12	Almuqbil et al. (18)	Y	N	Y	Y	Y	Y	N	Y	UC	Include
13	Aldakhil et al. (19)	Y	N	Y	Y	Y	Y	N	Y	UC	Include
14	Almalki et al. (20)	Y	N	Y	Y	Y	Y	UC	Y	N	Include
15	Almansour et al. (21)	Y	Y	Y	Y	Y	UC	Y	N	Y	Include
16	Alenezi et al. (22)	Y	Y	Y	Y	Y	Y	N	Y	UC	Include
17	Almusbah et al. (23)	Y	Y	UC	N	Y	Y	UC	N	UC	Include
18	Samannodi et al. (24)	Y	Y	Y	N	Y	Y	Y	Y	N	Include
19	Khatrawi and Sayed (25)	Y	Y	Y	Y	Y	Y	UC	Y	N	Include
20	Al-Qahtani et al. (26)	Y	Y	Y	Y	Y	UC	UC	Y	N	Include
21	Alhazza et al. (27)	Y	N	Y	Y	Y	Y	Y	Y	N	Include
22	Rajeh et al. (28)	Y	N	Y	Y	Y	Y	N	N	UC	Include
23	Al-Rasheedi et al. (29)	Y	N	Y	Y	Y	Y	Y	Y	N	Include

N, No; UC, Unclear; Y, Yes.

### 2.5 Data synthesis and analysis

Due to the anticipated heterogeneity in study designs and measures, a narrative synthesis approach was employed. A synthesis and summary were made of the findings on the prevalence and determinants of parental vaccine hesitancy toward COVID-19 vaccines in Saudi Arabia. Themes and patterns in the data were identified, and critical insights were reported.

### 2.6 Primary and secondary outcomes

The primary outcome of this systematic review is to assess the overall percentage of the participants willing/intending to vaccinate their children with COVID-19 vaccines. Secondary outcomes are to underline parents’ main concerns regarding COVID-19 vaccines and the primary misconceptions they might have that led them to such concerns.

## 3 Results

### 3.1 Search results

**Figure 1:**
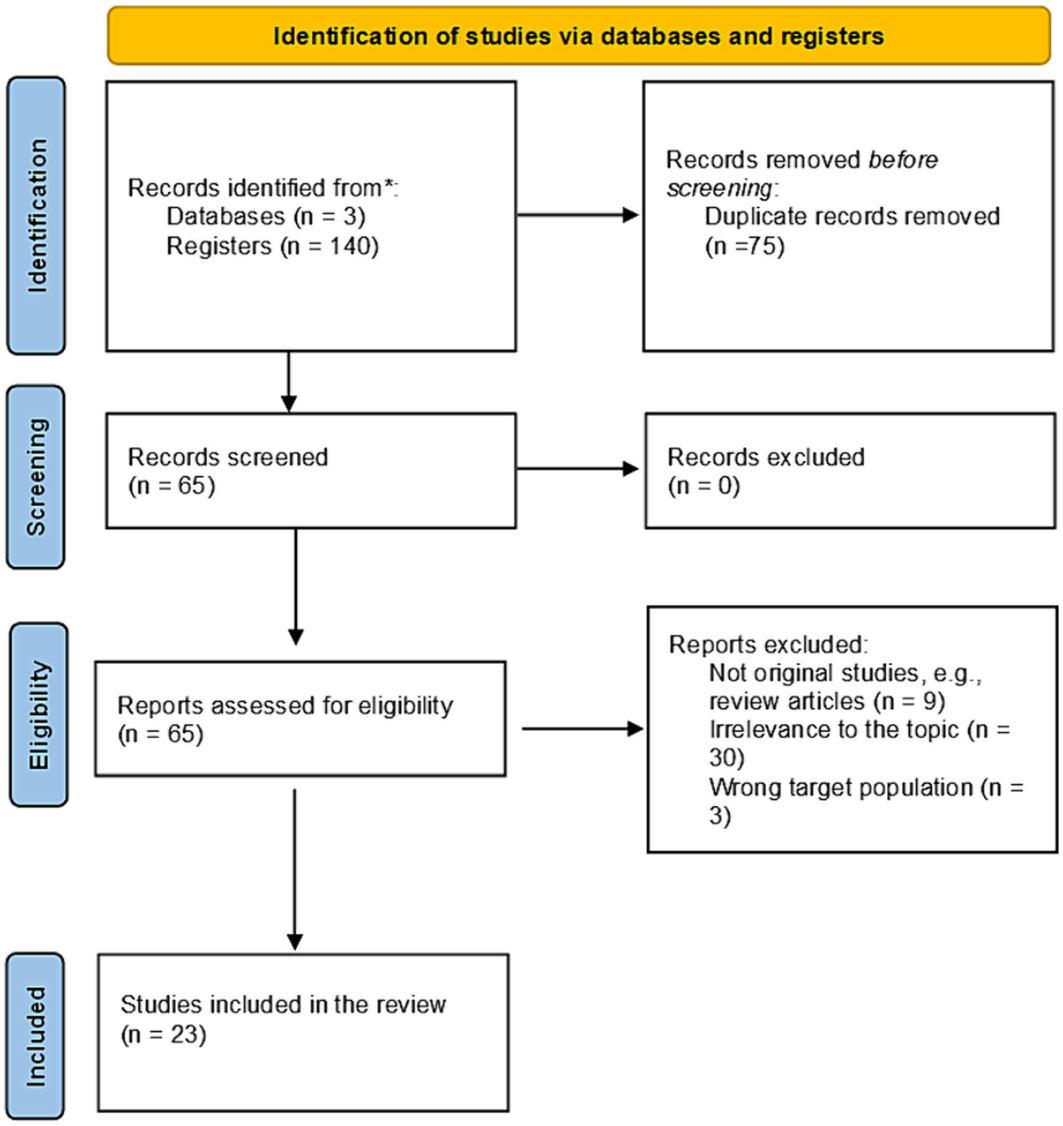
The Prisma flowchart has been adopted (31) for the study selection process in this systematic review. *n*, number of papers.

The article selection process followed the PRISMA guidelines (30), as demonstrated in Figure 1.

A total of 23 studies were included in this systematic review, which addressed either a proportion of the Saudi population, e.g., residents of a single city/region, or were conducted nationally. Over two-thirds (*n* = 16) of the conducted studies were on a national level. In contrast, ten studies were conducted locally, with five conducted in Riyadh, Saudi Arabia’s largest region.

A total of 20,926 participants took part in all the included studies; over two-thirds of the participants were women, with 37.27% being willing to vaccinate their children with COVID-19 vaccines. Only two studies did not clearly state the percentage of participants willing to vaccinate their children with COVID-19 vaccines (20, 28). The detailed characteristics of the included studies are described in Table 2.

**Table 2:** The characteristics of the studies included in this systematic review.

No.	References	Regional coverage	No. of participants	Willingness to vaccinate with COVID-19 vaccines
1	Temsah et al. (7)	National	3,167 (M: 1108, F: 2059)	47.6%
2	Altulaihi et al. (8)	Riyadh	333 (M: 167, F: 166)	54%
3	Aedh (9)	Najran	464 (M: 264, F: 200)	55.01%
4	Alzahrani and Alghamdi (10)	Taif City	301 (M: 93, F: 208)	89.37%
5	Ennaceur and Almohaithef (11)	National	379 (M: 191, F: 188)	44%
6	Shaati et al. (12)	National	1,463 (M: 623, F: 840)	55.4%
7	Al Saad et al. (13)	National	381^b^	54.8%
8	Al-Khlaiwi et al. (14)	National	1,304 (M: 342, F: 962)	46.1%
9	Alghamdi (15)	National	123 (M: 28, F: 95)	37.4%
10	Khan et al. (16)	Al-Jouf	444 (M: 155, F: 289)	42%
11	Alhuzaimi et al. (17)	Riyadh	873 (M: 297, F: 576)	48.2%
12	Almuqbil et al. (18)	Riyadh	699 (M: 581, F: 118)	41.6%
13	Aldakhil et al. (19)	Riyadh	270 (M: 0, F: 270)	43.77%
14	Almalki et al. (20)	National	4,135 (M: 784, F: 3351)	N/A^a^
15	Almansour et al. (21)	National	500 (M: 111, F: 389)	40.2%
16	Alenezi et al. (22)	National	1938^b^	46%
17	Almusbah et al. (23)	National	1,000 (M: 212, F: 788)	28.1%
18	Samannodi et al. (24)	National	581 (M: 225, F: 356)	63.9%
19	Khatrawi and Sayed (25)	National	344 (M: 140, F: 204)	37.8%
20	Al-Qahtani et al. (26)	Riyadh	528 (M: 118, F: 410)	34.2%
21	Alhazza et al. (27)	National	1,052 (M: 510, F: 542)	46%
22	Rajeh et al. (28)	Makkah	50 (M: 3, F: 47)	N/A^a^
23	Al-Rasheedi et al. (29)	Qassim	597 (M: 113, F: 484)	33.3%

a The percentage of the participants willing to vaccinate their children is not clearly stated, nor can it be calculated.

b The gender distribution of the participants was not specified in the study.

F, Female; M, Male.

### 3.2 The risk of bias appraisal

The studies included in this systematic review were analyzed using the JBI appraisal, the detailed breakdown of which is described in Table 1. The clarity of the study objectives was demonstrated in all studies except Altuhlaihi’s, and Ennaceur and Almohaithef’s studies. At least 12 (out of 23) studies clearly delivered the population representation, sample size adequacy, sampling technique description, data collection method clarity, measurement of variables, statistical analysis clarity, and identification of confounding factors. However, the appropriateness of prevalence estimates was not achieved clearly or unclear in most studies (19 out of 23).

### 3.3 Determinants of parental attitudes and hesitancy toward COVID-19 vaccination among their children

Several factors shaped the parental concepts and attitudes toward vaccinating their children against COVID-19. These factors could be broadly classified as demographic, socioeconomic, and health-related factors.

The studies included in this systematic review reported several demographic determinants influencing parents’ willingness, such as parental age, gender, nationality, and number of children. However, parental age and gender were the two most common factors behind parental hesitancy toward COVID-19 vaccines, described in 12 and 5 studies (out of 23), respectively. Mothers were less likely to vaccinate their children than fathers.

Similarly, the included studies reported several socioeconomic determinants of parental vaccine hesitancy, including but not limited to parental level of education, income level, employment status and the workplace nature. Out of these determinants, parental level of education and income, were described as influencing determinants in 12 and 6 studies, respectively. In most studies that indicated that the level of education contributed to parental vaccine hesitancy, the level of education directly correlated with parental willingness to vaccinate their children. In other words, the higher the education level, the more the parents were willing to vaccinate their children. Interestingly, only one study demonstrated an inverse relationship between education and parental willingness to vaccinate (10). Similarly, parental income level was directly correlated with parents’ willingness to vaccinate their children, i.e., parents with higher income were more likely to vaccinate their children than those with lower income. A less common factor that could be categorized along with the level of education and income is the parental job sector. Parents who are healthcare workers or with healthcare worker relatives were also more likely to vaccinate their children.

Three main factors were described in the context of health-related factors of parents’ hesitation toward vaccinating their children against COVID-19. These were previous parents’ vaccination with either COVID-19 or seasonal flu (influenza) vaccines, previous COVID-19 infection, and having children with chronic diseases. Previous vaccinations were indicated as crucial factors in 10 studies, in which parents’ worries about the vaccine’s potential safety were somewhat alleviated through their previous personal experience with them, which lacked any adverse effects (29). Similarly, the negative experience with COVID-19 and potentially its sequelae may have driven parents toward vaccinating their children. Additionally, parents are keen on protecting their children, which seems to be more pronounced when their children suffer from chronic conditions, making them liable to a severe form of COVID-19.

Some unique solitary factors were also found in the studies included in the systematic review. Altulaihi and colleagues described that the presence of extended family support was linked to parents’ willingness to vaccinate their children against COVID-19 (8). Other factors included the mutual involvement of both parents in decisions regarding the child’s care (16) and the type of COVID-19 vaccine to be given to the child (21).

### 3.4 Principal concerns and misconceptions affecting parental perception of COVID-19 for their children

The secondary outcome of this systematic review was to identify parents’ concerns about vaccinating their children against COVID-19 and the misconceptions associated with COVID-19 vaccination.

The potential and long-term side effects of the COVID-19 vaccines were reported in almost all studies included in the review as the parents’ most common concern before vaccinating their children. However, Saudi Arabia has had a long-standing national vaccination program, like other countries, which starts from a child’s birth. Hence, the concept of vaccination and the common vaccine adverse reactions such as fever and tenderness at the injection site are not new nor feared among parents. COVID-19 vaccines were considered a “rushed” product, which further deepened their concerns about the potential side effects of the vaccines. It was pretty interesting to have such a concern as 11 (out of 23) studies were published in 2022, and 6 studies were published in 2023, with eight studies indicating a lack of information on the safety of the vaccine, despite all the published reports indicating the safety of COVID-19 vaccines for children.

Another major concern that was indicated in 9 studies (of the 23) was about the vaccine’s effectiveness among children and whether it will protect children from the infection. Participants wondered whether the vaccine would be useful to their children and hoped to delay it until adulthood if given the choice (10). When Saudi Arabia extended its COVID-19 vaccination mandate, COVID-19 vaccines were available from several companies, e.g., Pfizer-BioNTech, AstraZeneca, and Moderna, which, at times, people could choose one of them. Interestingly, the availability of COVID-19 vaccines in Saudi Arabia and the choice given to parents were confusing to the parents as they did not know which would be a better choice for their children (10, 16, 21).

Several misconceptions have been identified among parents regarding vaccinating their children against COVID-19. The most common misconception stated in 10 (out of 23 studies) was that COVID-19 vaccines posed a risk to children, i.e., more dangerous than conventional vaccines. Samannodi and colleagues described that parents are worried about the development of blood clots as a result of the uptake of COVID-19 vaccines by their children (24). Other reported misconceptions believed to happen due to COVID-19 vaccines among children include genetic alteration and negatively affecting children’s future fertility (11, 12).

Another common misconception was that children were not at risk of contracting COVID-19 or a severe form of it, so vaccination was unnecessary. Such a misconception was stated in 10 studies included in the review.

As opposed to vaccination against COVID-19, some parents were more lenient toward adopting a natural approach and falsely believed that children’s natural immunity is better in combatting COVID-19 than vaccines. In 4 studies, parents stated that children’s natural immune system is sufficient to successfully tackle the infection, making the vaccination process redundant and carrying a risk of unknown side effects in the long term.

## 4 Discussion

The COVID-19 pandemic has triggered an unprecedented global response from the scientific community, leading to the rapid development and distribution of vaccines as a primary strategy to control the spread of the virus. However, the success of vaccination campaigns relies heavily on vaccine acceptance and uptake among the population. In Saudi Arabia, as in many other countries, vaccine hesitancy has emerged as a significant concern that threatens to impede progress toward achieving widespread immunity and ultimately ending the pandemic. This systematic review aims to comprehensively examine the landscape of parental vaccine hesitancy toward COVID-19 vaccines in Saudi Arabia in order to provide valuable insights that can inform public health strategies.

Vaccine hesitancy is not a novel phenomenon, but its significance has been amplified in the context of the COVID-19 pandemic. The World Health Organization (WHO) has identified vaccine hesitancy as one of the top ten threats to global health (32), given its global spread in other countries such as the United States (33), Canada (34), Japan (35) and Malaysia (36). However, the vaccine hesitancy among the Saudi population seems to exceed those in other countries (35) significantly.

The present systematic review demonstrated that a significantly low percentage of the participants, just over 37%, were willing to vaccinate their children against COVID-19. While such low willingness could threaten Saudi public health, it does not come across as a surprising finding. An earlier study by Almaghaslah and colleagues assessing vaccine hesitancy among Saudi participants showed that only 48% were willing to vaccinate against COVID-19 (37). Similarly, Almojaibel et al. showed in a recent study that only 49% of the participants received both doses of the COVID-19 vaccines (38), despite the mandated free and widely available vaccination as part of the Saudi measures taken against COVID-19 (1, 2, 39).

Saudi Arabia’s population is generally young compared to many developed countries, with a median age of 29 years in 2022 (40). This review identified parental age as one of the main factors described in 9 (out of 23) studies, influencing parents’ intention to vaccinate their children against COVID-19. However, this is hard to interpret as out of the 23 included studies, only three studies (16, 19, 21) included age as a continuous variable. The remaining studies categorized age into different age categories without providing a valid rationale for such a categorization. The age categorization has varied extensively between studies. For example, Al-Rasheedi and colleagues adopted an extensive age grouping, e.g., 18–24, 25–34, 35–44, 45–54, 55–64, 65 and older (29). In contrast, the study by Alhazza et al. adopted an arbitrary age cutoff, e.g., younger and older than 40 years old (27). Such variations prevent drawing a valuable conclusion on the effect of age on parental willingness to vaccinate their children with COVID-19 vaccines.

In this study, parents’ level of education was a determinant of their willingness to vaccinate their children. This finding was in line with what was described earlier by Al-Mohaithef and colleagues (41) and Al Naam et al. (42) on adults and their willingness to get vaccinated. On the other hand, the only study in the review by Alzahrani and Alghamdi (10) showed an inverse relationship between education and vaccine acceptance, i.e., the higher the education level, the less likely the parents to accept vaccination, which is unsurprising. Previous studies in Saudi Arabia have shown a similar inverse relationship (43, 44). Interestingly, these previously published studies were conducted exclusively on women, which further confirms the finding of this review that gender is also an essential determinant influencing vaccination decisions. In other words, mothers, as compared to fathers, were less willing to vaccinate their children. Such contradicting findings between parents’ educational level and their willingness to vaccinate their children require further studies to explain this phenomenon.

The potential side effects that children may suffer from COVID-19 vaccines were found to be the most common concern among parents. Understandably, a new vaccine may warrant concerns regarding its safety. Similar concerns were previously described among adults in Saudi Arabia (45). However, such concerns are not limited to Saudi Arabia and extend globally, such as Italy and the United States (46, 47). The parental concerns about COVID-19 vaccine safety and potential unknown side effects on their children could be justified. As previously discussed, adults also demonstrated a variable degree of hesitancy toward COVID-19 vaccinations. Hence, parents’ protective nature, especially mothers toward their children, would most likely enhance such hesitancy, driving them toward unwillingness to vaccinate their children against COVID-19. Noteworthily, such resistance starts from pregnancy to lactation throughout parenthood (48, 49).

COVID-19 vaccines have been hailed as crucial in controlling the global pandemic. Nevertheless, they have also become the subject of numerous misconceptions, fuelled in part by the rapid spread of misinformation on social media platforms. One of the most common misconceptions pertains to the speed at which these vaccines were developed. Some of the included studies expressed concerns that their expedited development may have compromised safety (24, 28). However, it is essential to note that the vaccines underwent rigorous clinical trials, adhering to established safety protocols (50). Another misconception involves the vaccines altering an individual’s DNA (11, 12). Messenger RNA (mRNA) vaccines like Pfizer-BioNTech and Moderna work by instructing cells to produce a harmless spike protein found on the surface of the virus, triggering an immune response. They do not integrate with an individual’s DNA (51). Such misconceptions have been previously reported among Saudi adults (52, 53). Social media platforms have played a pivotal role in spreading these misconceptions, both locally in Saudi Arabia and globally. Several studies included in this systematic review indicated that their source of information, including social media, was a key determinant in their willingness to vaccinate their children (7, 10, 12, 13, 21). A study by Pennycook and Rand (54) found that false information about COVID-19 is more likely to be shared on platforms like X, formerly known as Twitter, making it highly accessible to a broad audience. Additionally, social media has provided fertile ground for the proliferation of conspiracy theories and anti-vaccine sentiments (55). The spread of misinformation and its active role in increasing parental vaccine hesitancy extend beyond the borders of Saudi Arabia to other nations, e.g., Japan (56).

This systematic review of parental willingness to vaccinate their children against COVID-19 is the first in Saudi Arabia. The importance of this review stems from providing a much-needed reliable and reproducible summary of existing literature (57). Following the updated PRISMA guidelines (31) in this systematic review further validates its findings and facilitates reproducibility, a troubling challenge to today’s scientific community (58).

Despite the thorough effort in the conduction of this systematic review, several limitations should be taken into consideration when interpreting the findings of this review. The first observed limitation is the timing of the conducted studies included in this review. Only 3 (out of 23 studies) were conducted in the earlier phases of the pandemic, which would most likely reflect the true intention of parents to vaccinate their children against COVID-19. Most of the remaining studies were conducted after the Saudi government enforced a mass vaccination program on the population, despite its ethical challenges (59), during which some or most of the participants and their children were already vaccinated. For example, Almalki et al. (20) study included over 4,000 participants, of which over 94% have already vaccinated their children against COVID-19. Such an experience, the vaccination mandate, may have influenced their decision or intention toward vaccinating their child against COVID-19. Secondly, the primary outcome of this systematic review was to determine parents’ willingness to vaccinate their children with COVID-19 vaccines. However, most of the included studies were in fact assessing parents’ attitudes and hesitancy toward the vaccine. These studies either used validated tools, such as the Vaccine Hesitancy Scale (VHS) (60) and the Covid-19 Vaccine Hesitancy and Resistance in Saudi Arabia (CoV-HERSA) (61), or resorted to developing their own tools. Hence, such a variation in the results would be expected. The results would be consistent and can be easily compiled by healthcare policymakers if a single tool were used to assess parental vaccination willingness. Lastly, this systematic review included almost 21 thousand participants, representing about 0.06% of the total Saudi population of over 32 million (40). It is important to consider this small percentage when interpreting the results of this review, i.e., it could limit the generalizability of these results.

Future studies on parental vaccine hesitancy should address this review’s limitations. Future studies should be conducted as early as possible to understand the determinants of vaccine hesitancy among the public and their concerns and misconceptions. Furthermore, studies should also include subjects with special needs, e.g., subjects with disabilities (62).This will drive a better and tailored public health response to increase vaccination uptake. Furthermore, the Ministry of Health, in collaboration with academics from around the country, should work together using a validated instrument to compile a large dataset. This dataset would most likely collect a representative sample of the Saudi population. Finally, healthcare policymakers should make use of this systematic review, as well as similar studies published on the adult population, to prepare and respond for future outbreaks, either on a local scale, such as the Middle East Respiratory Syndrome (MERS) (63), or on a global scale such as COVID-19 pandemic.

## 5 Conclusion

The mass COVID-19 vaccination programs in Saudi Arabia were vital in containing the infection; however, they were also associated with vaccine hesitancy and resistance among the public. This review provides a vital and much-needed summary of the current determinants of vaccine hesitancy among parents toward vaccinating their children against COVID-19. Understanding the determinants that influence such hesitancy, the public significant concerns, as well as the common vaccine-related misconceptions are key to public health policymakers. Knowing and addressing these factors will allow us to provide timely scientific recommendations to the public, enhancing their acceptance significantly (64). After all, the vaccine is only effective when people take it, and such hesitancy may hinder the official efforts to curb the infection.

## Data availability statement

The original contributions presented in the study are included in the article/supplementary material, further inquiries can be directed to the corresponding author.

## Author contributions

AS: Conceptualization, Data curation, Formal analysis¸ Funding acquisition, Investigation, Methodology, Project administration, Resources, Software, Supervision, Validation, Visualization, Writing – original draft, Writing – review & editing.
